# Re-evaluation of boundaries of Streptococcus mitis and Streptococcus oralis and demonstration of multiple later synonyms of Streptococcus mitis, Streptococcus oralis and Streptococcus thalassemiae: description of Streptococcus mitis subsp. carlssonii subsp. nov. and emended description of Streptococcus mitis

**DOI:** 10.1099/ijsem.0.006704

**Published:** 2025-03-11

**Authors:** Mogens Kilian, Hans-Christian Slotved, Kurt Fuursted, Adonis D’Mello, Hervé Tettelin

**Affiliations:** 1 Department of Biomedicine, Aarhus University, Skou building 1115-139A, C. F. Møllers Allé 6, DK-8000 Aarhus C, Denmark; 2 Department of Bacteria, Parasites and Fungi, Statens Serum Institut, Copenhagen, Denmark; 3 Department of Microbiology and Immunology, Institute for Genome Sciences, University of Maryland School of Medicine, Baltimore, Maryland, USA

**Keywords:** ANI values, classification of *Streptococcus *species, dDDH values, evolution, later synonyms, Mash distances, phylogenomic and comparative genomic analyses, *Streptococcus mitis*, *Streptococcus oralis*

## Abstract

The commensal species *Streptococcus mitis* and *Streptococcus oralis* are genetically diverse to a degree that challenges traditional definitions of species. This causes automatic identification based on DNA sequences or cellular extract profiles problematic. Based on an initial analysis of 266 genomes, we subjected a subset of 100 representative genomes to detailed phylogenetic, pairwise distance and gene pattern analyses. *S. mitis* and *S. oralis* constitute a continuum of clones that are genetically unique. To recognize most isolates as separate species is biologically and practically meaningless. We recommend bending the proposed similarity borders to accommodate the biological reality. Accordingly, we conclude that *Streptococcus toyakuensis*, *Streptococcus chosunensis*, *Streptococcus gwangjuensis*, *Streptococcus humanilactis* and *Streptococcus hohhotensis* are later heterotypic synonyms of *S. mitis*. Type strains of effectively but not validly published ‘*Streptococcus shenyangsis*’, ‘*Streptococcus symci*’ and ‘*Streptococcus vulneris*’ belong in *S. mitis. Streptococcus parapneumoniae* and *Streptococcus nakanonensis* are later synonyms of *Streptococcus thalassemiae. Streptococcus downii* is a later synonym of *Streptococcus oralis* subsp. *dentisani*, and the type of ‘*Streptococcus halitosis*’ belongs in *Streptococcus oralis* subsp. *tigurinus*. The genome sequence of the type of the recently proposed ‘*Streptococcus bouchesdurhonensis*’ is based on a mixed culture. Phylogenetic results and the pattern of presence/absence of accessory genes support the distinction of two subspecies of *S. mitis*, i.e. *Sreptococcus mitis* subsp*. mitis* subsp. nov. (type strain is NCTC 12261^T^) and *Sreptococcus mitis* subsp. *carlssonii* subsp. nov. (type strain is SK608=CCUG 55085^T^=LMG 33510^T^). The special population structure of the *Streptococcus mitis–pneumoniae–pseudopneumoniae–thalassemiae* complex renders automated classification of isolates based on average nucleotide identity or digital DNA–DNA hybridization values problematic. As an alternative, for initial taxonomic assignment, we present a whole-genome phylogeny-based method that enables phylogenetic comparison of new isolates in the context of a set of 117 well-characterized reference strains assigned to the Mitis/Sanguinis group.

## Introduction

Automated identification of bacteria based on DNA sequences, from single loci to near-complete genomes, or on cellular extract profiles has revolutionized the working processes in clinical microbiology. Dependent on comprehensive reference databases and a biologically satisfactory taxonomy, these methods offer rapid and reliable assignments for most bacterial species. However, some groups of bacteria still present problems.

One example is the Mitis group of the genus *Streptococcus*, which includes both important human pathogens and commensals. The term Mitis group was introduced by Kawamura *et al.* [1] based on phylogenetic analysis of 16S rRNA gene sequences. Initially, the group included six species. Subsequently, new species were described and added to the Mitis group, while others were shown to be later synonyms of already existing species as revealed by analyses of more comprehensive DNA sequence data [2]. According to phylogenetic analyses of complete genome sequences, Richards *et al.* [3] restricted the term Mitis group to eight species (*Streptococcus mitis*, *Streptococcus pneumoniae*, *Streptococcus pseudopneumoniae*, *Streptococcus oralis*, in addition to the more distant *Streptococcus infantis*, *Streptococcus peroris*, *Streptococcus australis* and *Streptococcus parasanguinis*) and introduced the new term, the Sanguinis group, to accommodate *Streptococcus sanguinis*, *Streptococcus gordonii* and *Streptococcus cristatus.* As reported by Jensen *et al.* [2], population genetic studies revealed that *S. oralis* includes *Streptococcus oralis* subsp. *oralis*, *Streptococcus oralis* subsp. *tigurinus*, *Streptococcus oralis* subsp. *dentisani* (formerly ‘*Streptococcus mitis* biovar 2’) and at least one genomosubspecies. More recently, numerous proposals for new species belonging to the Mitis group were based on studies of a single isolate. These include *Streptococcus toyakuensis* [4], *Streptococcus thalassemiae* [5], *Streptococcus chosunensis* [6], *Streptococcus gwangjuensis* [7], *Streptococcus humanilactis* [8] and the effectively but not yet validly published names ‘*Streptococcus shenyangsis*’, ‘*Streptococcus symci*’, ‘*Streptococcus vulneris*’ and ‘*Streptococcus bouchesdurhonensis*’ [9,12]. Although belonging to the Mitis group, their relationship to existing species is not entirely clear.

Based on genome sequence analyses, *S. pneumoniae* presents a coherent cluster of clones with limited DNA sequence diversity of core genes. In contrast, the commensal species *S. mitis*, *S. oralis* and *S. infantis*, as presently defined, show a degree of genetic diversity that challenges traditional taxonomic principles [2,13,15]. A recently reported multilocus sequence typing (MLST) scheme for *S. mitis* failed to identify clonal clusters within the population [16] apart from strains that derived from the same samples (see below). Collectively, these studies demonstrate a surprising genetic uniqueness of virtually every isolate of *S. mitis*. Furthermore, isolates of *S. mitis* may express varying combinations of proteins and capsular polysaccharides that normally are associated with virulence in *S. pneumoniae* [17,18].

We reported evidence for the evolutionary scenario that the populations of *S. mitis* and *S. pneumoniae* evolved from a common ancestor similar to today’s pneumococcus. As a result of an evolutionary bottleneck, presumably caused by a shortage of potential hosts, the lineages currently assigned to *S. mitis* eliminated genes that challenged their host, thus securing immune tolerance, sustained colonization and mainly vertical transmission between hosts. The few clones that evolved into today’s *S. pneumoniae* potentiated their ability to survive by genetic plasticity and horizontal transmission among human hosts, a strategy that subsequently proved successful in parallel with the increasing size and density of the human population as reflected in the recent burst of the pneumococcal population [13,19]. The plasticity of the pneumococcal genome is enhanced by the import of genes, including genes in the capsular polysaccharide synthesis (Cps) locus and mutated penicillin-binding proteins conferring resistance, from other pneumococci and the large genetic pool constituted by *S. mitis* and other commensal Mitis group streptococci. Comprehensive analysis demonstrated that up to 8% of genes in genomes of *S. pneumoniae* are *S. mitis* alleles [19]. This evolutionary model is consistent with the variable numbers of functional or truncated ‘*S. pneumoniae* virulence genes’ in *S. mitis* strains and the long-term sequence diversification of * S. mitis* clones taking place within separate lineages of hosts [13,19]. According to DNA–DNA reassociation and sequence divergence values, the genetic diversity within *S. mitis*, *S. oralis* and *S. infantis* has reached a level that renders virtually every isolate a separate species by the traditional taxonomic standards [13,17]. Furthermore, the described evolutionary scenario explains the sometimes problematic distinction of *S. mitis* and *S. pneumoniae* in the laboratory. Part of this problem is *S. pseudopneumoniae* [20], which is an intermediary between *S. mitis* and *S. pneumoniae* and often gives rise to identification issues [2,14]. In a comprehensive study of misidentified clinical isolates of Mitis group streptococci, Sadowy *et al.* [14] concluded that no identification method based on single loci or even multilocus assays like Multilocus Sequence Analysis (MLSA) is without problems.

Prompted by a recent clinical isolate that proved difficult to identify, we revisited the definition of the species *S. mitis* and its relationship to neighbouring species including the numerous novel species recently described.

## Methods

### New clinical isolate

The isolate PNC-2022-0275 was recovered in 2022 from a sputum sample from a 72-year-old male cultivated on 5% horse blood agar (SSI). Matrix-assisted laser desorption ionization time-of-flight mass spectrometry (MALDI-TOF) analysis and tests for optochin susceptibility and bile solubility were performed as described [21]. Whole-genome sequencing was performed as previously described [22]. Briefly, genomic DNA was extracted using a DNeasy Blood and Tissue Kit (QIAGEN, Hilden, Germany), and fragment libraries were constructed using a Nextera XT Kit (Illumina, San Diego, CA, USA) followed by 150 or 250 bp paired-end sequencing on the MiSeq or NextSeq 550 platforms (Illumina, San Diego, CA, USA), respectively, according to the manufacturer’s instructions. The paired-end reads were filtered using Bifrost v2.0.8 (https://github.com/ssi-dk/bifrost) and *de novo* assembled using the SKESA assembler (v.2.2) [23]. The assembled genome was examined at the TYGS (Type (strain) Genome Server) at https://tygs.dsmz.de [24].

The PNC-2022-0275 assembled genome was combined with 265 assembled genomes available at Genbank as of December 2022. These assemblies include genomes that were listed as *S. mitis*; the related species that are discussed in this manuscript, including the more recently released *S. thalassemiae* [5] and ‘*S. bouchesdurhonensis*’; and the following selected representative reference genomes: 6 of *S. oralis* subsp. *oralis*, 5 of *S. oralis* subsp. *dentisani*, 5 of *S. oralis* subsp. *tigurinus*, 1 of *S. oralis* genomosubsp. 1, 3 *S. pseudopneumoniae*, 13 *S. pneumoniae*, the type of *Streptococcus downii* and 1 *S. infantis* (Table S1, available in the online Supplementary Material).

### Nucleotide identity

All genome sequences were quality-checked for completeness and contamination using the CheckM software v1.2.3 [25]. An average nucleotide identity (ANI)-like pairwise distance matrix was generated with Mash v2.3 [26], and this matrix was used to generate a neighbour-joining tree using megacc v10.0.5 [27].

Actual ANI pairwise calculations were performed between all possible pairs among each of a subset of 100 genomes using FastANI v1.33 [28] with default parameters. Pairwise digital DNA–DNA hybridization (dDDH) analyses on the same subset of 100 genomes were performed as described by Meier-Kolthoﬀ *et al.* [29] on the TYGS server (https://tygs.dsmz.de/).

### Phylogenetic analysis of aligned core genomes

A whole-genome multiple nucleotide alignment was generated for two subsets (100 and 82 genome sequences, see below) using Mugsy v1.2.3 [30]. Core locally collinear blocks of the resulting alignments in MAF (Multiple Alignment Format) format, i.e. sequences present in all 100 or 82 genomes, respectively, were concatenated and converted to fasta format for phylogenetic analyses.

Phylogenetic and molecular evolutionary analyses were conducted using mega v11 (https://www.megasoftware.net) [31] and SplitsTree4 v4.14.6 (https://uni-tuebingen.de/fakultaeten/mathematisch-naturwissenschaftliche-fakultaet/fachbereiche/informatik/lehrstuehle/algorithms-in-bioinformatics/software/splitstree/) [32] using default parameters.

### Mapping of selected genes

We performed a search for selected genes that we previously identified as being specific or near specific for *S. pneumoniae* or particular subgroups of *S. mitis* [17]. Their presence was analysed by blastn search against selected assemblies using cut-off values ≥60% nt sequence identity over ≥40% of the sequence length as previously implemented [17].

## Results

### Properties of a clinical isolate

The isolate PNC-2022-0275 showed short chains of elongated Gram-positive cocci under the microscope, greening around colonies on blood agar (‘*α*-haemolysis’), and no catalase activity. Based on an inhibition zone of 28 mm around an optochin disc, the isolate was rated as optochin-sensitive. The bile solubility test, which, after incubation for 10 min at 36 °C, determines the difference of absorbance of bacteria in a test tube containing 10% sodium deoxycholate versus a blank control, showed the value 2.7. We previously demonstrated a range of 2.3–3.4 for *S. pneumoniae* strains and 0–2.55 for strains of *S. mitis* (21). The MALDI score suggested that the isolate belonged to *S. mitis*, whereas the MALDI-TOF peak evaluation [33] supported the identity as *S. pneumoniae* [21].

Genome sequencing of strain PNC-2022-0275 resulted in a sequence of 1 895 869 nt (GenBank accession number JAZKTX000000000; GCA_036785505.1) distributed on 110 contigs of sizes ranging from 90 155 to 79 nt (N50 : 42,980). The G+C content was 40.0 mol%. The total number of genes was 2242, the number of protein-coding genes (CDS) was 2107 and the number of pseudogenes was 86. The completeness was 99.82%, and the contamination was 0.4.

Upload of the assembly sequence on the Pathogenwatch database (https://pathogen.watch/genomes/) [34] resulted in the identification of *S. pneumoniae* (*P*-value 0). Based on the sequence, the database identified resistance to erythromycin, tetracycline, trimethoprim, sulfamethoxazole and co-trimoxazole. According to penicillin-binding protein (PBP) analysis [35], predicted MICs (microgram per millilitre) were penicillin, 2; amoxicillin, 2; cefuroxime, >2.

The *S. pneumoniae* MLST (Multilocus Sequence Type) database (https://pubmlst.org/organisms/streptococcus-pneumoniae) only recognized two out of seven target loci in the uploaded genome. The recently described *S. mitis* MLST database (https://pubmlst.org/bigsdb?db=pubmlst_smitis_seqdef) detected the loci of all seven MLST genes, *accA*, *gki*, *hom*, *oppC*, *patB*, *rlmN* and *tsf*, but only recognized one out of the seven, *patB* as a complete match (allele 67). Manual sequence blast showed 94.0–97.1% identity to allele 1 of the remaining six loci as query sequence. An attempt to identify the isolate using the ribosomal MLST approach [36] resulted in *S. pneumoniae* with 60% support and *S. mitis* and *S. pseudopneumoniae* each with 20% support.

Analysis of the genome sequence on the TYGS platform (https://tygs.dsmz.de) yielded a phylogenetic tree with type strains of ‘*Streptococcus chosunense*’ (*Streptococcus chosunensis corrig*. Lim *et al*. 2024), ‘*Streptococcus gwangjuense'* (*Streptococcus gwangjuensis corrig*. Park *et al*. 2024), ‘*S. shenyangsis*’, ‘*S. humanilactis*’ (*Streptococcus humanilactis* Guo et al. 2024), *S. toyakuensis*, ‘*S. symci*’, ‘*S. vulneris*’ and *S. downii* as the closest neighbours, and the overall conclusion is ‘Potential new species’.

### Phylogenetic analyses

For an initial analysis, we extracted the seven genes used in the *S. pneumoniae* MLST system (*aroE*, *gdh*, *gki*, *recP*, *spi*, *xpt* and *ddl*) and performed phylogenetic analyses with corresponding sequences of 60 representatives of *S. pneumoniae*, *S. pseudopneumoniae*, *S. mitis*, *S. oralis* and *S. infantis* [19] in mega v11. In all analyses, the PNC-2022-0275 clustered among *S. mitis* strains and not *S. pneumoniae* (not shown).

For a more comprehensive analysis of the clinical isolate in relation to *S. mitis* and related species, we selected 221 genome sequences listed as *S. mitis* in the GenBank database. Sequences of designated types were always included, while sequences of questionable quality (size <1 800 000; contamination >5%) were excluded. An additional 44 genomes/assemblies were included: 13 *S. pneumoniae*, 3 *S. pseudopneumoniae*, 6 *S. oralis* subsp*. oralis*, 5 *S. oralis* subsp*. tigurinus*, 5 *S. oralis* subsp*. dentisani*, 1 *S. oralis* genomosubsp. 1, *Streptococcus* sp. SK643 and type strains of *S. chosunensis*, *S. downii*, *S. gwangjuensis*, * S. humanilactis*, *S. infantis*, ‘*S. shenyangsis*’, ‘*S. symci*’, *S. toyakuensis*, ‘*S. vulneris*’ and ‘*S. bouchesdurhonensis*’ (Fig. S1). Quality check of the genome sequences showed that the majority were more than 98% complete with less than 2% contamination (Table S1). Notable exceptions were the designated types of ‘*S. bouchesdurhonensis*’ and ‘*S. symci*’, which showed 80 and 16% contamination, respectively.

Based on a Mash ANI-like pairwise distance matrix [26], a neighbour-joining tree was constructed in mega v11. The comprehensive tree of 266 strains is shown in Fig. S1. Although the tree lacks the resolution power of the trees based on aligned genomes (see below), it reveals 16 strains listed as *S. mitis* in the Genbank database that, according to their position in the tree, belong to other well-established taxa (Table S1). In addition, based on GenBank information, 49 genomes represented re-sequencing or re-annotations of the same strain. The type strain of *S. mitis* NCTC 12261/SK142^T^ alone was represented by six entries in the database. The analysis furthermore revealed several pairs or groups of up to 23 genome sequences showing 100% identity. Without exception, these represent isolates from the same sample or groups of 2, 6 or 23 genomes of isolates from the genitals of a single couple of sexual partners [37]. Thus, they appear to represent repeated analyses of the same clone.

From the tree in Fig. S1, we selected 100 genome sequences that include all relevant lineages from each of the *S. mitis* subclusters together with the reference genomes from other species listed above and excluded redundant sequences and genome sequences that were of questionable quality. Actual pairwise ANI and dDDH calculations were performed between all possible pairs among the 100 genomes using FastaANI v1.33 [28] with default parameters and the TYGS platform [24], respectively (Table S2). Submitting the 100 genome sequences for identification on the TYGS server yielded the conclusion of ‘potential new species’ for all apart from the genome sequences of designated type strains.

**Fig. 1.**
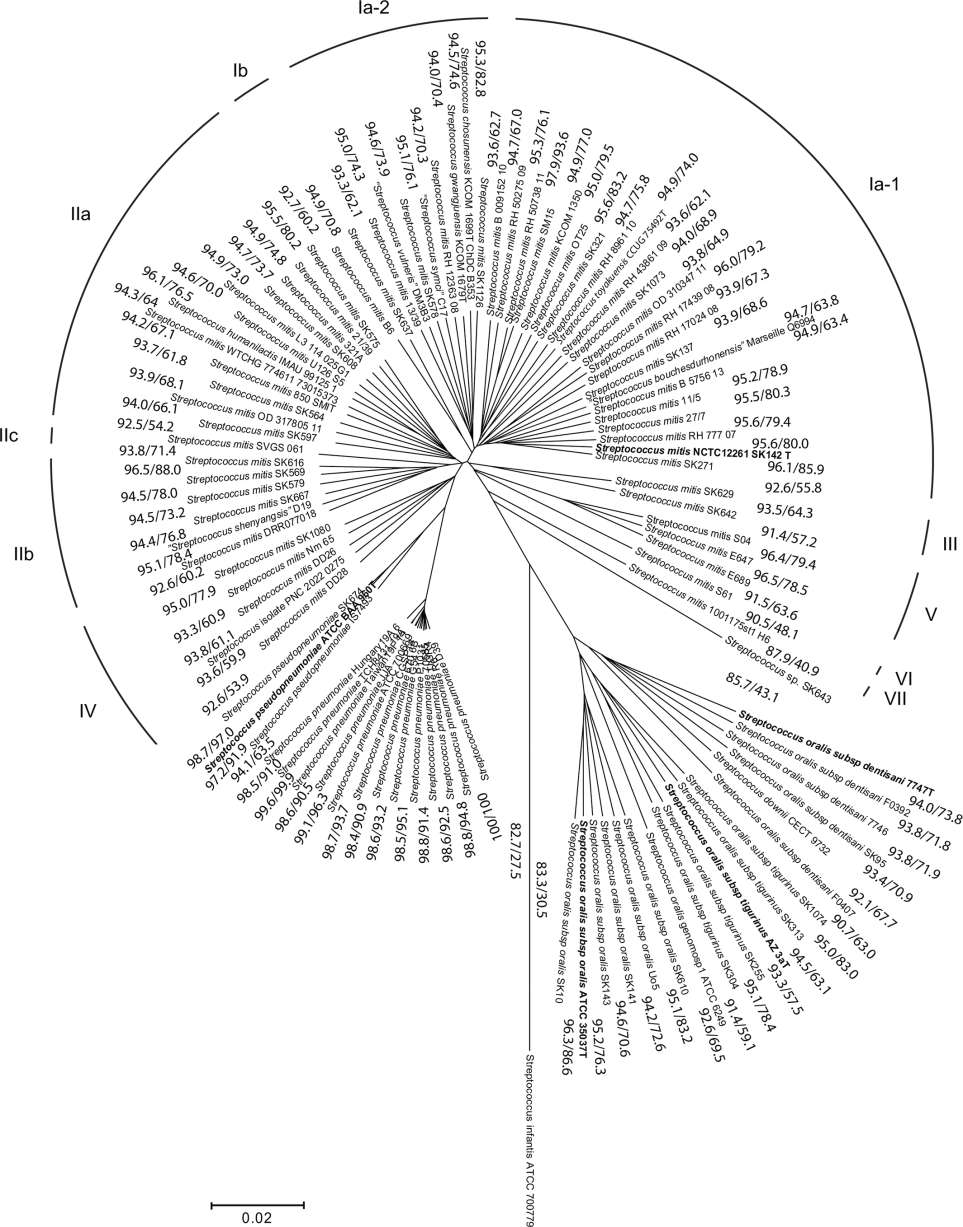
Phylogenetic relationship of 100 *Streptococcus* strains inferred by the minimum evolution method conducted in mega v11 [38] using a core sequence of 453 542 bp. The tree is drawn to scale, with branch lengths in the same units as those of the evolutionary distances used to infer the phylogenetic tree. The evolutionary distances were computed using the maximum composite likelihood method [27] and are in the units of the number of base substitutions per site. Bootstrap analysis based on 500 replications showed that all nodes were supported by bootstrap values >82. ANI/dDDH (***d*_6_**) values are shown between each neighbouring pair of strains. Each cluster of strains previously assigned to *S. mitis* is labelled in Roman numerals. Their identity, revised according to this study, is discussed in the text.

The 100 genome sequences were subjected to more detailed phylogenetic analysis based on a sequence alignment performed by the Mugsy algorithm [30]. The core segments of the 100 aligned genomes encompassed an average of 435 289 nt. Fig. 1 shows a phylogenetic tree constructed in mega v11 [38] using the minimum evolution algorithm. The tree demonstrates a tight cluster of the 13 *S. pneumoniae* strains; a less dense but distinct cluster of 3 *S. pseudopneumoniae* strains, distinct lineages representing the type of *S. infantis* and the unclassified *Streptococcus* sp. SK643; and a distinct cluster of genetically divergent lineages representing *S. oralis* clearly separated into the 3 subspecies *oralis*, *dentisani* and *tigurinus* and the singular strain of genomosubsp. 1. The cluster of *S. oralis* subsp*. dentisani* includes the designated type of *S. downii* [39].

The remaining 62 strains formed a continuum of distinct lineages of singletons previously assigned to *S. mitis* in addition to designated type strains of 8 recently proposed species, *S. chosunensis*, *S. gwangjuensis*, ‘*S. shenyangsis*’, *S. humanilactis*, * S. toyakuensis*, ‘*S. symci*’, ‘*S. vulneris*’ and ‘*S. bouchesdurhonensis*’, and the clinical isolate PNC-2022-0275. Some of the lineages form clusters, yet with limited distinction between them. One exception is a cluster of three strains labelled *S. mitis* S04, E647 and E689 from two yet unpublished studies of endocarditis and endophthalmitis, respectively. For strains included also in our previous study [17], the clustering in the tree is identical to that previously noted based on selected genes with discriminatory power. Accordingly, the clusters will be labelled Ia, Ib, IIa, IIb, IIc and III, in addition to two new clusters labelled IV and V and singleton strains VI and VII in Fig. 1. PNC-2022-0275 belonged to cluster IV. Cluster V is the mentioned well-separated cluster of three strains S04, E647 and E689.

**Fig. 2.**
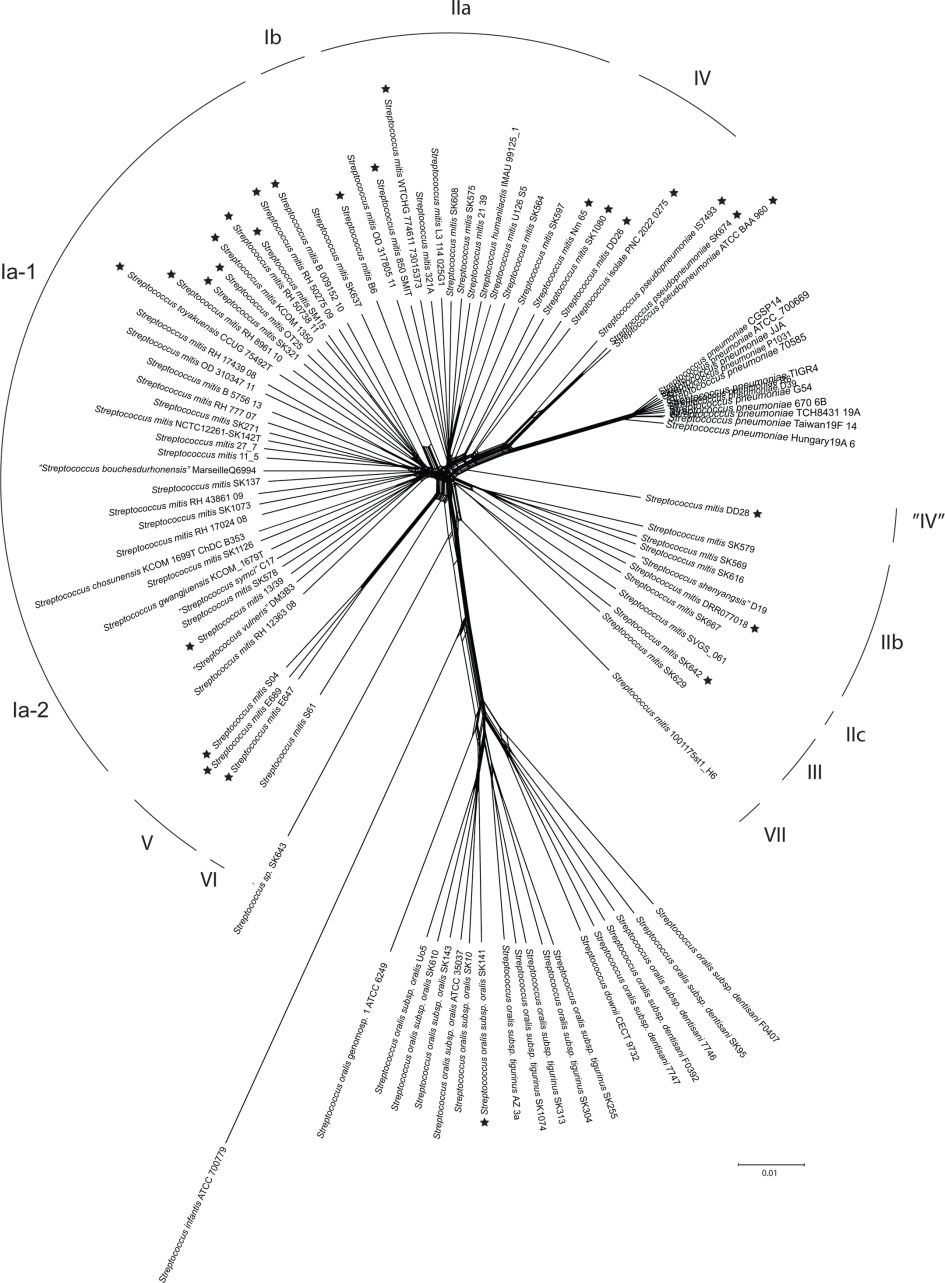
Evolutionary relationships of 100 genomes inferred from SplitsTree analysis of the same core alignment as in Fig. 1. The tree confirms the clustering pattern of both *S. mitis* and *S. oralis* demonstrated in Fig. 1. The 12 strains constituting *S. mitis* cluster IIa, together with strains of cluster IV, show a continuum of similarities and signs of genetic interaction with *S. pseudopneumoniae* and *S. pneumoniae*. However, according to this tree, cluster IV is not monophyletic. The six strains constituting cluster IIb and the single strain of cluster IIc appear more distinct from cluster IIa than in the tree in Fig. 1. The type strains of *S. toyakuensis*, *S. chosunensis*, *S. gwangjuensis*, *S. humanilactis*, ‘*S. shenyangsis*’, ‘*S. symci*’ and ‘*S. vulneris*’ are integral parts of the major clusters of *S. mitis.* The tree confirms that the three strains of cluster V constitute a coherent and distinct group. Strains marked with a star lack the capsular biosynthesis locus.

The overall clustering pattern is confirmed in the tree constructed in SplitsTree (Fig. 2), which identifies signs of recombination events between individual genomes [32]. According to SplitsTree, 30 *S. mitis* strains, including the type NCTC 12261, form a cluster of diverse lineages (clusters Ia-1 and Ia-2) without clear subdivisions. According to the analysis, inter-strain recombination has played a limited role in the evolution of clusters Ia-1 and Ia-2. The 12 strains constituting cluster IIa, together with strains of cluster IV, show a continuum of similarities and signs of genetic interaction with *S. pseudopneumoniae* and *S. pneumoniae*. However, according to this tree, cluster IV is not monophyletic. In particular, strain DD28 is well-separated from the remaining strains in cluster IV. The six strains constituting cluster IIb and the single strain of cluster IIc appear more distinct from cluster IIa than in the tree in Fig. 1. Again, the type strains of *S. chosunensis*, *S. gwangjuensis*, ‘*S. shenyangsis*’, *S. humanilactis*, *S. toyakuensis*, ‘*S. symci*’ and ‘*S. vulneris*’ are integral parts of the mentioned sections of *S. mitis.* Although belonging to *S. mitis*, the relationship of clusters Ib and III to the two main clusters I and II is inconclusive. The tree confirms that the three strains of cluster V constitute a coherent and distinct group.

**Fig. 3.**
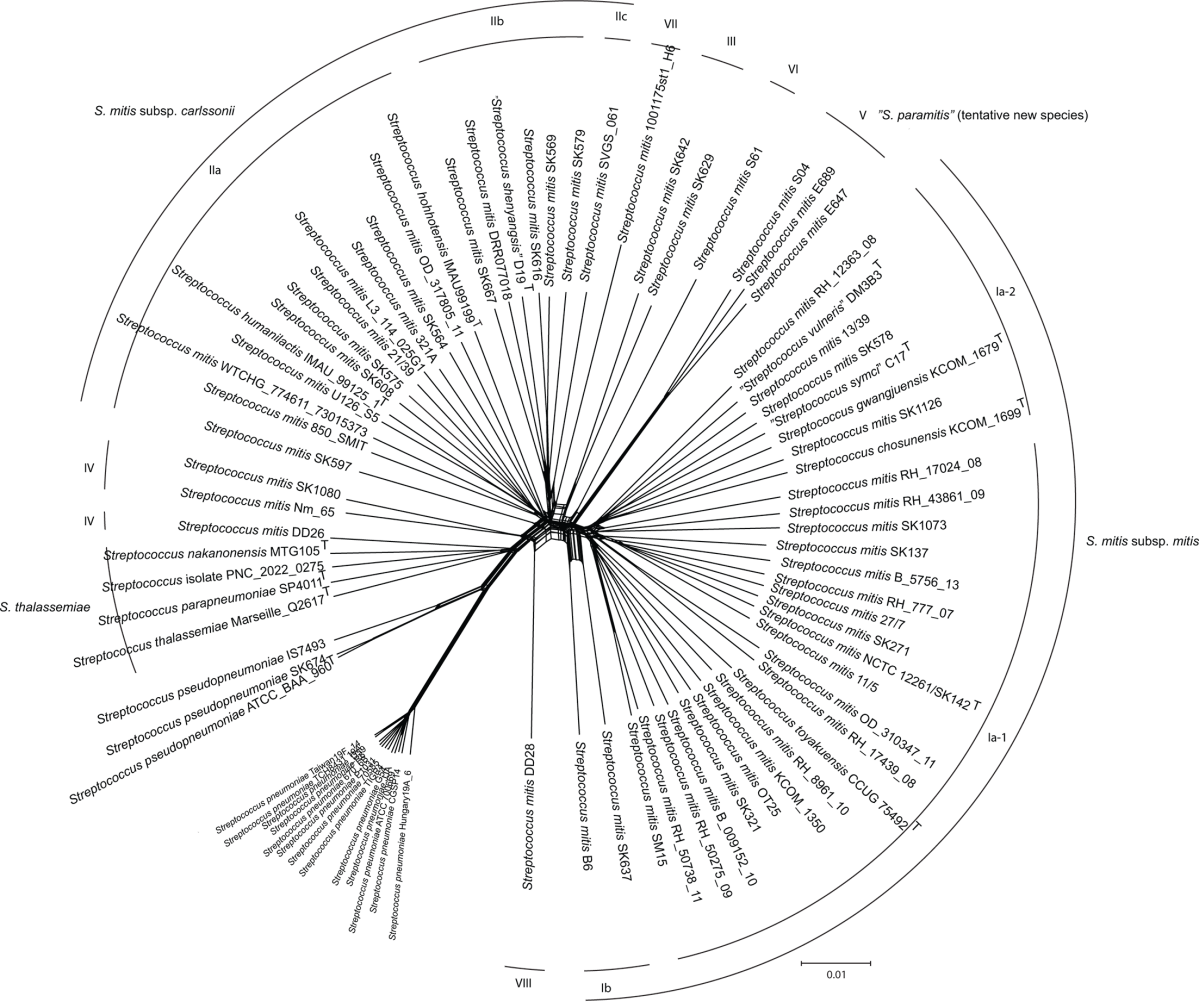
Phylogenetic relationship of 82 strains of *S. mitis*, *S. thalassemiae*, *S. pneumoniae* and *S. pseudopneumoniae* inferred by the SplitsTree algorithm [32]. The analysis was based on a core sequence of 1 106 042 bp. Bootstrap analysis based on 500 replications showed that all nodes were supported by bootstrap values >90. According to the comprehensive results of this study, including the gene analysis data in Table 2, clusters Ia-1, Ia-2 and Ib are proposed as *Streptococcus mitis* subsp. *mitis*, clusters IIa, IIb and IIc are proposed as *Streptococcus mitis* subsp. *carlssonii.* The tree confirms that cluster IV of Fig. 1 lacks coherence but indicates that four strains (*S. thalassemiae* Marseille Q2617, *S. parapneumoniae* SP4011, *S. nakanonensis* MTG105 and our clinical isolate PNC_2022_0275) form a coherent and distinct cluster. The remaining strains of cluster IV are separated into several distinct lineages including strain DD28, which is separate (‘cluster VIII’). Strains in clusters III, IV and VIII are considered non-classified members of * S. mitis*. Cluster V is recognized as a tentative candidate species ‘*Streptococcus paramitis*’. The strains S61 (cluster VI) and 1001175st1_H6 (cluster VII) are not considered members of *S. mitis* as they are genetically distant from that population.

To further optimize the clustering pattern of *S. mitis* complex strains, we performed an alignment of 82 strains including the * S. mitis* complex strains together with strains of *S. pneumoniae* and *S. pseudopneumoniae*. The genome sequence of the types of four species reported during the evaluation and revision of this manuscript, i.e. *S. thalassemiae* [5], *Streptococcus parapneumoniae* [40], *Streptococcus nakanonensis* [41] and *Streptococcus hohhotensis* [42], were included in this dataset (Table S1). Excluding strains of *S. oralis* and *S. infantis* and the questionable ‘*S. bouchesdurhonensis*’ (see below) more than doubled the size of the core genome to 1 045 079 nt, thus providing improved resolution. The SplitsTree is shown in Fig. 3. The tree further supports the separation of *S. mitis* complex strains into two major clusters (Ia-1 and Ia-2, and IIa and IIb, respectively) in addition to the well-separated cluster V. Among a few lineages consisting of one to two strains (‘clusters IIc, III, VI and VII’) is also strain DD28 (‘cluster VIII’). Like in the tree shown in Fig. 2, cluster IV, containing PNC-2022-0275, is less monophyletic than indicated by the analysis in Fig. 1. The designated type of *S. thalassemiae* here forms a reasonably tight cluster with our clinical isolate PNC-2022-0275 and the types of the recently described *S. parapneumoniae* and *S. nakanonensis.* This cluster is distinct from the strains DD26, Nm-65 and SK1080. The type of the proposed *S. hohhotensis* clusters with cluster IIa of *S. mitis* strains.

Several unique strains raise the question of optimal taxonomic affiliation (Figs 3 and S2). In all trees, the strains SK643 and 1001175st_H6 (cluster VII) are unique and show a lower similarity to the type of *S. mitis* than to *S. pneumoniae* and * S. pseudopneumoniae* (dDDH (*d*_6_)=57.7 and 51.5%, respectively) indicating that they do not belong in the species *S. mitis*. Likewise, strain SVGS_061 (cluster IIc) is only 57.8% similar (dDDH (*d*_6_)) to the type NCTC12261, and the association with *S. mitis* is questionable. Another unique strain, S61 (cluster VI), surprisingly is 65.4% similar to the type of *S. mitis* according to dDDH (*d*_6_) determination, which is well within the range of similarity levels for *S. mitis*.

### Average nucleotide identity (FastANI), Mash distances and dDDH

The triangular matrix of all pairwise Mash ANI-like distances between 270 genome sequences is shown in Table S3 and the complete dataset of FastANI, Mash distances and dDDH values for every pair of the subset of 100 strains is shown in Table S2. The table includes an addendum with dDDH data relevant to the taxonomic assignment of recently added types of *S. parapneumoniae*, *S. nakanonensis* and *S. hohhotensis.*

A correlation analysis was conducted to test the overall performance of the three methods used for pairwise genetic similarities, dDDH (*d*_6_), Mash distances and FastANI. The results presented in Fig. S3 illustrate a significant degree of correlation (*R*≥0.92) between the results of the three tests. The correlation is superior in pairwise comparisons of the most closely related strains, whereas FastANI values below 87% appear to lack the discriminatory power of the two other methods.

The pairwise FastANI and dDDH (*d*_6_) values for each of the neighbouring pairs are included in the phylogenetic tree in Fig. 1. Using the *S. mitis* boundaries discussed above, the range of FastANI values for *S. mitis* is from 96.1 to 92.6%, values that confirm the pattern of continuum in the population of *S. mitis* with no clear-cut breaks between clusters. The same is the case for the parallel dDDH (*d*_6_) values for neighbouring *S. mitis* pairs in Fig. 1, i.e. 85.9–60.2%. The inter-cluster values between cluster I and cluster II are 92.7 (ANI) and 60.2% (dDDH (*d*_6_)), respectively.

The full range of dDDH (*d*_6_) values for pairs of the *S. mitis* type (NCTC 12261^T^) and any of the other strains assigned to * S. mitis* (clusters Ia-1, Ia-2, Ib, IIa, IIb, IIc and IV) is 85.9–59.4% (Table S2). The ranking of dDDH (*d*_6_) values of all strains compared to *S. mitis* NCTC 12261^T^ and a representative of clusters II, strain SK608, respectively, is shown in Fig. S2. The graphs further support the continuum of similarities within the *S. mitis* population. The data support the overall clustering pattern in Figs1, 2  3, but there are notable exceptions. The majority of cluster Ia-1 are, as expected, most similar to strain NCTC 12261^T^. However, some cluster IIa and IIb strains are more similar to strain NCTC 12261 ^T^ than some cluster Ia-1 and Ia-2 strains. Likewise, the cluster II reference strain SK608 is most similar to cluster IIa strains but appears closer to most cluster Ia strains than to cluster IIb strains. Clusters were defined using the phylogeny shown in Figs1, 2  3 which is based on a core genome multiple alignment. Values such as dDDH and fastANI are calculated on a pairwise basis and, therefore, use genome sequence information from both the core and the accessory genomes. Thus, it is not surprising to find that, for instance, genomes from cluster Ia are not all contiguous in Fig. S2.

Confirming the observations from the phylogenetic trees, the corresponding intra-cluster ANI values for cluster V pairs are 96.4–96.5% (79.4–78.5% for dDDH (*d*_6_) values) and values below 92.3 (63.6) to all its closest neighbours (including *S. mitis* NCTC 12261) (Tables 1 and S2).

**Table 1.** Summary of intra- and inter-taxon dDDH (p6) (upper triangle) and FastANI values (lower triangle) for 100 genomes*

	[1]	[2]	[3]	[4]	[5]	[6]	[7]	[8]	[9]	[10]	[11]	[12]	[13]
[1] *S. mitis* (incl. Ia-b, IIa-c, III, IV, VIII)	62.2 (47.1–93.6) 93.3 (91.8–97.9)					59.6 (49.4–68.0)	42.6 (33.4–51.0)	42.5 (34.4–50.4)	43.3 (34.6–51.0)	42.1 (33.4–49.8)	53.2 (46.9–59.3)	53.6 (46.6–59.2)	57.0 (46.6–66.3)
[2] *S. mitis* subsp. *mitis* (cluster Ia-b)			60.1 (50.4–68.9)	58.0 (50.8–68.2)	57.2 (47.1–66.1)	61.0 (54.1–68.0)	44.0 (36.3–51.0)	44.0 (37.3–50.4)	44.7 (37.6–51.0)	43.5 (36.3–49.8)	52.6 (46.9–58.3)	52.8 (46.6–59.2)	55.8 (46.6–62.6)
[3] *S. mitis* subsp. *carlssonii* (cluster IIa-c)		92.9 (91.8–93.8)		59.8 (54.5–65.1)	59.3 (48.1–68.0)	58.8 (55.3–63.4)	41.4 (36.2–46.8)	41.2 (38.4–46.8)	42.2 (38.3–45.7)	40.9 (36.2–44.5)	54.1 (49.4–58.2)	54.6 (50.1–57.3)	58.2 (52.4–61.5)
[4] *S. mitis* (cluster III)		92.4 (91.9–92.8)	92.4 (92.2–92.7)		57.6 (50.6–63.3)	55.0 (52.3–57.8)	40.1 (36.3–43.0)	39.8 (37.7–43.0)	40.5 (37.4–42.8)	39.7 (36.3–43.0)	51.0 (47.6–54.4)	52.4 (50.7–54.5)	55.6 (52.1–59.3)
[5] *S. mitis* (cluster IV)		92.8 (91.9–93.5)	93.1 (92.0–93.9)	92.3 (91.9–92.7)		54.7 (49.4–58.6)	38.7 (33.4–44.5)	38.8 (34.4–44.5)	39.4 (34.6–43.4)	38.0 (33.4–42.7)	55.1 (52.1–59.3)	55.5 (53.9–57.3)	62.7 (60.3–66.3)
[6] ‘*S. paramitis*’ (cluster V) tentative new species	92.0 (91.1–92.7)	92.2 (91.6–92.7)	91.8 (91.3–92.3)	91.3 (91.1–91.5)	91.6 (91.2–91.8)		42.8 (38.4–47.7)	42.9 (40.2–47.7)	43.2 (41.0–45.5)	42.7 (38.4–45.0)	53.3 (50.6–56.3)	52.8(51.8–53.6)	54.4 (53.7–56.8)
[7] *S. oralis* (incl. three subsp. and two genomosubsp.)	86.8 (86.1–87.5)	86.9 (86.2–87.5)	86.7 (86.1–87.3)	86.7 (86.3–87.0)	86.6 (86.1–87.1)	86.4 (86.0–87.0)	64.1 (50.2–86.6) 92.5 (90.3–96.3)				39.5 (36.2–43.2)	37.0 (34.1–39.4)	38.5 (34.7–40.9)
[8] *S. oralis* subsp. *oralis*	86.9 (86.3–87.5)	87.0(86.4–87.5)	86.7 (86.3–87.3)	86.8 (86.4–87.0)	86.7 (86.4–87.1)	86.4 (86.2–87.0)			59.4 (55.8–64.7)	62.7 (54.0–70.3)	38.7 (36.2–41.9)	36.8 (35.2–38.7)	38.2 (36.4–40.9)
[9] *S. oralis* subsp. *dentisani*	86.9 (86.2–87.4)	87.0 (86.2–87.4)	86.8 (86.3–87.2)	86.6 (86.3–86.9)	86.6 (86.2–86.9)	86.4 (86.2–86.6)		91.9 (91.5–92.5)		60.7 (50.2–67.9)	40.5 (37.6–43.2)	37.8 (35.9–39.4)	39.1 (35.8–40.9)
[10] *S. oralis* subsp. *tigurinus*	86.9 (86.2–87.4)	87.0 (86.3–87.4)	86.7 (86.3–87.1)	86.7 (86.3–86.9)	86.7 (86.2–87.1)	86.4 (86.2–86.6)		92.9 (92.4–93.5)	91.7 (91.1–92.0)		39.7 (36.7–43)	36.5 (34.1–38.1)	38.0 (34.7–40.9)
[11] *S. pneumoniae*	91.8 (91–92.9)	91.6 (91.0–92.3)	91.9 (91.2–92.4)	91.3 (91.2–91.4)	92.7 (92.5–92.9)	90.8 (90.7–91.1)	86.6 (86.2–86.9)	86.6 (86.4–86.9)	86.5 (86.2–86.8)	86.6 (86.3–86.9)	91.7 (87.8–99.9) 98.6 (98.2–99.6)	65.4 (62.8–68.2)	58.4 (55–61.7)
[12] *S. pseudopneumoniae*	92.2 (91.5–93.2)	92.0 (91.5–92.6)	92.3 (91.5–92.9)	91.6 (91.5–91.7)	93.1 (92.8–93.2)	91.1 (91.0–91.2)	86.4 (86–86.9)	86.5 (86.3–86.8)	86.4 (86.1–86.9)	86.5 (86.2–86.9)	94.4 (94.2–94.6)	93.6 (91.0.9–97) 97.7 (97.1–98.8)	63.1 (62.2–63.9)
[13] *S. thalassemiae*	92.8 (91.9–94.0)	92.6 (91.9–93.2)	92.9 (92.0–93.7)	92.1 (92.0–92.1)	93.9 (93.8–94.0)	91.5 (91.4–91.7)	86.6 (86.1–86.9)	86.6 (86.4–86.9)	86.6 (86.2–86.7)	86.5 (86.3–86.8)	93.2 (93.0–93.3)	93.7 (93.6–93.8)	71.6 (66.7–78.0) 94.8 (94.5–95.1)

* Mean value and value range in brackets. Individual pairwise values are found in Table S2. Intra-species comparisons on the diagonal ([1] to [1], [7] to [7], [11] to [11], [12] to [12], [13] to [13]) list two sets of values: dDDH values on the first line and FastANI values on the second line.

By comparison, the overall range of ANI similarity values for *S. oralis* is 96.3 to 93.3; equivalent dDDH values are 86.6 to 57.5. The ANI value range for *S. oralis* subsp. *oralis* is 94.2–96.3 and for *S. oralis* subsp. *tigurinus* is 93.3–95.1. Four of the strains assigned to *S. oralis* subsp. *dentisani* and the type of *S. downii* form a cluster with pairwise ANI values of 93.4–94.0. The exception is * S. oralis* subsp. *dentisani* F0407 for which the pairwise ANI values to all other strains of the cluster are only 92.2–92.7.

The ANI values for *S. pneumoniae* (98.6; range 98.1–99.6 excluding the pair D39 and R6, which derive from the same strain) and *S. pseudopneumoniae* (97.7; range 97.0–99.0) confirm that they are genetically coherent species and concur with the proposed cut-off ANI values and dDDH values for members of the same species [43,44]. As shown also by the inter-species ANI values in Table 1, *S. pneumoniae* and *S. pseudopneumoniae* are well-separated from neighbouring species.

### Accessory genes

The mean total number of CDS in the *S. mitis* strains included in the study is 1904. The corresponding figure for *S. pneumoniae* and *S. pseudopneumoniae* is 2124 and 2120, respectively, in agreement with the reductive evolution of the *S. mitis* genome [13,19]. By comparison, the mean CDS number in *S. oralis* genomes is 1880. Using our Sybil database, developed to characterize the evolution of Mitis group streptococci and potential virulence factors [17] which includes a subset of the strains of this study, we determined the proportion of protein-encoding genes conserved in the genomes of the individual species. The conserved gene core for 20 *S. mitis* genomes was 1138 genes, which is 60% of the genes that most strains of the species possess. The corresponding figures for 16 *S. oralis* strains and 13 *S. pneumoniae* strains are 1139 genes (60%) and 1359 genes (64%), respectively. By comparison, the figure for nine of the significantly larger genomes of *Escherichia coli* is 3050 corresponding to 55–60% of the genes that most strains of that species possess [43].

ANI and dDDH values are based on genes and inter-gene sequences shared between two strains, which, for the 20 strains also included in our previous study [17], range from 1337 to 1555 genes, depending on the phylogenetic closeness of strains. Thus, several hundred genes may vary between a pair of strains of the same species and are not included in the similarity data or the phylogenetic analyses based on core genome alignments.

*S. mitis* is known for its homogeneous reactions in standard phenotypic tests previously used to identify the Mitis group species, i.e. negative reaction in tests for aesculin and arginine hydrolysis, urease, extracellular dextran or levan, fermentation of inulin, sorbitol and mannitol and neuraminidase. In accordance with all other species belonging to the Mitis group, they are alpha-haemolytic (i.e. H_2_O_2_-producers).

To further test the validity of the phylogenetic clusters detected by core genome analyses, we determined the presence or absence of (1) the selected genes previously determined to be specific for either *S. pneumoniae* or *S. mitis* [17] and (2) genes previously shown to be variably present in *S. mitis* [17]. The latter included genes constituting the capsular biosynthesis locus. The results presented in Table 2 show many variations within clusters, but, in addition, some clear-cut differences between clusters.

**Table 2.** The presence of selected genes demonstrated by blast search (identity >60%, coverage >40%) in strains of individual phylogenetic clusters

Gene	*S. mitis* complex cluster												*S.* *pneu* *-moniae*	*S.* *pseudo-pneu* *-moniae*	*S.* *thalas* *-semiae*	*S. oralis* subsp.		
	Ia-1	Ia-2	Ib	IIa	IIb	IIc	III	IV	V*	VI	VII	VIII				*oralis*	*denti* *-sani*	*tigurus*
	*N*=22	*N*=8	*N*=2	*N*=12	*N*=6	*N*=1	*N*=2	*N*=3	*N*=3	*N*=1	*N*=1	*N*=1	*N*=13	*N*=3	*N*=4	*N*=6	*N*=5	*N*=5
Capsule biosynthesis locus (cps)	13/22	7/8	1/2	11/12	5/6	+	1/2	–	–	+	?	–	+	–	–	5/6	+	+
SP_1154: IgA1 protease, zmpA†	2/22	+	–	11/12	5/6	+	1/2	–	–	–	–	–	+	+	3/4	+	–	–
SP_0117/SPD_0126: PspA	–	–	–	–	–	–	–	–	–	–	–	–	+	–	Fragm.	–	–	–
SP_0314: hyaluronidase	–	–	–	–	–	–	–	+	–	–	–	+	+	–	2/4	–	–	3/5
SP_0322: glucuronyl hydrolase	–	–	–	–	–	–	–	+	–	–	–	+	+	–	2/4	–	–	3/5
SP_1923: pneumolysin	–	–	–	2/12	–	–	–	+	–	–	–	+	+	+	x2	–	–	–
SP_1937: autolysin	7/22	–	1/2	4/12	–	–	–	+	–	–	–	+	+	+	+	–	–	–
SP_1992: surface protein ‘Xisco’	–	–	–	–	–	–	–	1/3	–	–	–	–	+	–	+	–	–	–
SP_2021 : 6-phospho-β-glucosidase	–	–	–	–	–	–	–	–	–	–	–	–	+	–	–	–	–	–
SP_0159/SMSK564_RS10275: divalent metal cation transporter	1/22	–	–	11/12	5/6	+	–	+	–	+	+	+	+	+	+	+	+	+
SP_0207: conserved hypothetical protein	4/22	3/8	+	1/12	2/6	–	+	2/3	1/3	+	+	+	+	+	+	5/6	3/5	4/5
SP_0242–4: ABC transporter	19/22	–	–	+	4/6	+	+	+	+	+	+	+	+	+	+	2/6	–	–
SP_0296: hypothetical protein	–	1/8	+	2/12	1/6	–	1/2	–	–	–	–	+	4/13	–	–			
SP_0648: *β*-galactosidase	2/22	+	–	–	+	+	–	1/3	+	–	–	–	+	+	–	+	4/5	+
SP_0666: conserved hypothetical protein	21/22	3/8	1/2	5/12	1/6	–	+	+	2/3	–	–	+	8/13	+	2/4	–	–	–
SP_0667: putative surface protein	+	4/8	+	8/12	+	+	+	+	+	+	+	+	+	+	+	–	–	–
SP_0918–22: spermidine synthase locus	+	+	1/2	8/12	5/6	+	+	+	+	+	–	+	+	+	+	+	4/5	4/5
SP_1300: hypothetical protein	3/22	+	+	–	+	+	–	1/3	–	+	+	+	+	+	2/4	–	–	–
SP_1494: hypothetical protein	1/22	–	1/2	1/12	–	–	–	–	–	–	–	–	+	+	–	–	–	–
SP_1495: hypothetical protein	–	–	–	–	–	–	–	–	–	–	–	–	+	–	–	–	–	–
SP_1500: aa ABC transporter	20/22	3/8	+	9/12	5/6	+	+	+	2/3	+	+	+	+	+	+	–	+	–
SP_1504: sialidase NanA	–	–	–	–	–	–	–	–	–	–	–	–	+	+	+	3/6	+	+
SP_1573: lysozyme lytC	–	–	–	+	5/6	+	+	+	–	–	+	+	+	+	+	–	–	–
SP_1704/6: ABC transporter	–	–	–	9/12	–	–	–	1/3	–	–	–	–	+	+	3/4	–	–	–
SP_1740–1: addiction module toxin/antitoxin	6/22	4/8	1/2	9/12	3/6	+	1/2	+	+	–	–	–	10/13	–	2/4	5/6	–	2/5
SP_1945: putative membrane protein	–	–	–	11/12	+	–	1/2	+	–	–	+	+	+	2/3	+	–	–	–
SP_2032–8: hexulose-6-phosphate isomerase locus	8/22	–	1/2	6/12	3/6	+	–	–	–	–	–	+	+	–	2/4	–	–	1/5
smi_0862: acetyl transferase	+	+	+	–	–	–	–	–	1/3	+	–	–	–	–	–	–	–	–
smi_2042–4: nicotinamide locus	+	+	+	1/12	–	+	+	+	+	–	+	+	–	+	–	+	+	+
SM12261_1609: competence- regulated protein	+	+	+	–	–	–	–	–	+	–	–	–	–	–	–	–	–	–
SK1126_1577: nucleotide tyrosine protein phosphate	3/22	+	–	5/12	+	+	–	–	–	–	–	–	+	–	–	2/6	2/5	–
SK608_1252: platelet aggregating factor	2/22	–	–	11/12	+	+	+	–	–	–	+	–	–	–	3/4	–	–	–
SK608_1273: fucolectin-related protein	–	–	–	+	+	+	+	+	–	–	+	+	–	+	+	–	–	–
SMSK564_RS09145: hypothetical protein	–	–	–	+	+	+	1/2	+	–	–	+	+	+	+	+	–	–	–
SMSK564_RS11490: membrane protein	–	–	1/2	11/12	+	–	+	+	–	+	+	+	+	+	+	–	2/5	1/5

* Cluster V is tentatively named ‘*Streptococcus paramitis*’ in the text.

† The *zmpA* (IgA1 protease) gene in strains of *S. mitis* clusters Ia-1 and Ia-2 is located in the same genomic context as in strains of *S. oralis*, while in *S. mitis* strains of clusters IIa, IIb, III and IV, the gene is located as in strains of *S. pneumoniae*.

The specificity of some genes for *S. pneumoniae* is confirmed (i.e. SP_0117/SPD_0126: PspA; SP_2021 : 6-phospho-β-glucosidase; SP_1495: hypothetical protein). However, other genes that often are considered specific to *S. pneumoniae* were present in some * S. mitis* strains, most notably strains of cluster IV (i.e. SP_0314: hyaluronidase; SP_0322: glucuronyl hydrolase; SP_1923: pneumolysin; SP_1937: autolysin; and SP_1992: surface protein ‘Xisco’). The hyaluronidase cluster genes (SP_0314 and SP_0322) were present in the clinical isolate PNC_2022-0275 as well as in three out of five strains of *S. oralis* subsp. *tigurinus* (Table 2). A fragment (30%) of the *pspA* gene (SP_0117) was present in PNC_2022-0275. In general, fragments of ‘*S. pneumoniae* genes’ were frequently detected in strains of the *S. mitis* complex, particularly in cluster IV strains. Interestingly, all four strains assigned to *S. thalassemiae* according to the tree in Fig. 3, each have two copies of the pneumolysin gene *ply*.

Complete capsular biosynthesis loci that included four regulatory genes and genes encoding flippase (*wzx*), polymerase *(wzy*) and various combinations of glycosyl transferases and carbohydrate-modifying enzymes were detected in the majority of the 100 genomes (Table 2). The size of the locus located between *dexB* and *aliA* ranged between ~16 000 and 29 000 nt. Interestingly, in *S. mitis* strain WTCHG 7746111 (cluster IIa), the complete Cps operon was located in the locus harbouring the *zmpA* gene in other cluster II strains and strains of *S. pneumoniae*. According to blast analyses of similarity, many *S. mitis* strains appear to express capsular polysaccharides identical to recognized pneumococcal capsular serotypes as previously reported [18,45]. One strain of *S. mitis* cluster Ia-1 expresses a serotype 10A capsule and two strains of cluster Ia-2 serotype 36. Among the cluster IIa strains, two strains of serotype 5 and one strain each of serotypes 1, 19C, 43 and 45 were detected. Cluster IIb included three strains of serotype 45 and one strain of serotype 18. Strain SK629 of cluster III appears to express a serotype 33D capsule. The remaining strains with a complete Cps locus showed limited similarity to known pneumococcus serotypes. The lack of a Cps locus corresponded to phylogenetic positions and included all strains belonging to *S. pseudopneumoniae*, *S. thalassemiae*, *S. mitis* clusters IV and V and a subset of cluster Ia-1 (Fig. 2). The only *S. oralis* strain lacking a Cps locus was SK141. The span between *dexB* and *aliA* varied between ~4000 and 7500 bp in all Cps-negative strains.

The distinction of *S. mitis* clusters I (a-b) and II (a-c) is supported by the presence or absence of selected genes, though rarely without exceptions. As shown in Table 2, cluster I strains possessed the genes encoding acetyl transferase (smi_0862), nicotinamide ADP-ribose pyrophosphatase, YjhB, nicotinamide mononucleotide transporter, nicotinamide-nucleotide adenylyltransferase, NadR (smi_2042–4) and competence-regulated protein (SM12261_1609). Conversely, strains of clusters II and III possessed the genes *zmpA* (IgA1 protease), SP_0159/SMSK564_RS10275 (divalent metal cation transporter), SMSK564_RS09145 (hypothetical protein), SMSK564_RS11490 (membrane protein), SP_1573 (lysozyme, LytC), SP_1945 (putative membrane protein), SK608_1273 (fucolectin-related protein) and SK608_1252 (platelet aggregating factor). The first six are present also in *S. pneumoniae* and *S. pseudopneumoniae* (Table 2) supporting the closer evolutionary relationship of cluster II strains to those species. This is further supported by the fact that, in cluster II strains, the *zmpA* gene is located in the same genomic context as in *S. pneumoniae* and *S. pseudopneumoniae*, while in the occasional *zmpA*-positive cluster I strains, the gene is located in the same genomic contexts as in *S. oralis* subsp. *oralis* (Table 2).

The gene presence/absence data offer limited support for the subdivisions within clusters I and II.

### ‘*Streptococcus bouchesdurhonensis*’

According to the core-genome-based phylogenetic analysis, the type of ‘*S. bouchesdurhonensis*’ clusters with the type of *S. mitis* cluster Ia-1. Unexpectedly, the sequence representing ‘*S. bouchesdurhonensis*’ included most of the genes considered specific to *S. pneumoniae* and *S. pseudopneumoniae*. This is in contrast to all other strains clustering with the type of *S. mitis* (cluster Ia-1). Genes that were present also in strains of the Ia-1 cluster were detected in two copies in the ‘*S. bouchesdurhonensis*’ genome, one with sequence identity to strains of *S. pseudopneumoniae* and the other with sequence identity to strains of *S. mitis* cluster Ia-1. This made us suspect that the sequence is a mixture of two strains, presumably strains of *S. mitis* and *S. pseudopneumoniae*. Three additional observations support this assumption: (1) the sequence representing the type of ‘*S. bouchesdurhonensis*’ shows a dichotomy of closest ANI values, i.e. to the type of * S. pseudopneumoniae* (96.1) and to that of *S. mitis* (95.3); (2) the cumulative size of the ‘*S. bouchesdurhonensis*’ sequence deposited in the Genbank database amounts to 3 766 235 bp, which is far above the range for streptococci. The 99 other genome sequences included in the final analysis range from 1 807 960 to 2 245 615 bp; and (3) the genome sequence shows a CheckM contamination value of 80%.

## Discussion

A bacterial species is essentially a coherent population of clones that is distinct from other populations [46]. The classic challenge in prokaryotic taxonomy is what level of population diversity corresponds to the categories in the taxonomic hierarchy, i.e. genus, species or subspecies. An overall guideline has been that members of a species should show 70% or higher DNA–DNA reassociation values [47]. To allow for the experimental error inherent to relevant methods, it has been argued that a gap between 60 and 70% DNA–DNA hybridization values seems more biologically realistic to embrace clear-cut clusters of organisms [48]. Studies evaluating newer methods have shown that the corresponding ANI value range for conserved genes is 94–96% [43,49] and that the digital analogy of dDDH is 70% [29]. The practical importance of these boundaries is augmented by the increased use of online identification of bacterial isolates based on genome sequences. Using genome sequences of designated types as a reference, the databases will determine if the query genome is sufficiently similar to be assigned to a recognized species. However, they do not take into account the differences in the population diversity of individual species. This problem is clearly illustrated by the fact that the TYGS facility, which works very well with most other bacteria, was able to identify only designated type strains and strains of *S. pneumoniae* and *S. pseudopneumoniae* included in our study. All other strains resulted in the response ‘potential new species’.

Within the genus *Streptococcus*, some species like *S. pneumoniae*, *S. pyogenes*, *S. canis* and *S. mutans* show a high degree of genetic homogeneity well within the recommended borders. Other species including *S. mitis*, *S. oralis*, *S. infantis* and *S. suis* show a degree of diversity that exceeds the proposed DNA–DNA homology and ANI/dDDH boundaries [2,13, 15, 50]. According to Zhou *et al.* [50], *S. mitis* encompasses at least 44 distinct 95% ANI clusters judged by comprehensive pangenome analyses. The significant sequence divergence within the *S. mitis* and *S. oralis* clusters is confirmed by the present study (Table S2). Presumably, exclusive niche-specific adaptation, limited DNA exchange and mainly vertical transfer between hosts shaped this diversity [17].

The overall diversity picture for the *S. mitis* complex (excluding cluster V) is a continuum of pairwise ANI and dDDH values in the range of 96.1–92.6 (inter-cluster values 94.0–92.5) and 85.9–60.2 (inter-cluster values 82.8–60.2), respectively, which do not make separation into distinct taxa obvious (Fig. 1). The dDDH values for the type of *S. mitis* NCTC 12261^T^ and each of the strains labelled * S. mitis* form an unbroken continuum between 85.9 and 59.4% as shown in Fig. S3. As a result, the majority of isolates would qualify as separate species according to the proposed boundaries for prokaryotic species, which explains the numerous proposals for new species. During the first 8 months of 2024, eight new species of viridans streptococci were proposed, all based on a single isolate. The *S. mitis* cluster strains in our study include designated types of the species *S. toyakuensis*, *S. humanilactis*, *S. chosunensis*, *S. gwangjuensis* and *S. hohhotensis*, as well as the effectively published but not yet validated, ‘*S. shenyangsis*’, ‘*S. symci*’, ‘*S. vulneris*’ and ‘*S. bouchesdurhonensis*’*.* Likewise, the relatively dense cluster that includes the recently reported species *S. thalassemiae* also included the proposed types of * S. parapneumoniae* and *S. nakanonensis* (Fig. 3).

For the *S. oralis* cluster, the pairwise range of ANI values is 96.3–93.3, but with significantly lower values (90.7–92.6) between the three recognized subspecies and the genomosubspecies 1. The *S. oralis* subsp. *dentisani* strain F0407 seems to be distinct from the remaining strains of *S. oralis* subsp. *dentisani* including the type (closest neighbour 92.1). Accordingly, strain F0407 will be referred to as *Streptococcus oralis* genomosubspecies 2.

Recognizing each isolate of *S. mitis* and *S. oralis* as separate species is neither practical nor biologically meaningful. Therefore, we recommend bending the proposed borders to accommodate the biological reality of these groups of bacteria. Accordingly, we conclude that *S. toyakuensis*, *S. chosunensis*, *S. gwangjuensis*, *S. humanilactis* and *S. hohhotensis* are later synonyms of * S. mitis* and that *S. downii* is a later synonym of *S. oralis* subsp. *dentisani*. Likewise, the validation of the effectively published names ‘*S. shenyangsis*’, ‘*S. symci*’ and ‘*S. vulneris*’ is not supported by our findings as the designated types are part of *S. mitis*. The type of recently proposed species *S. thalassemiae* forms a distinct cluster with designated types of *S. parapneumoniae* and *S. nakanonensis*, in addition to our clinical isolate PNC-2022-0275 (Fig. 3). The cluster possesses many of the properties of *S. pneumoniae* and *S. pseudopneumoniae* including the pneumolysin, autolysin and the surface protein ‘Xisco’ (Table 2). Like strains of *S. pseudopneumoniae*, these strains lack a capsular polysaccharide biosynthetic locus, but all strains have duplicate copies of the pneumolysin (*ply*) gene. According to these observations, *S. thalassemiae* deserves recognition as a separate species, while *S. parapneumoniae* and *S. nakanonensis* are later synonyms due to the priority of publication of *S. thalassemiae*.

‘*S. bouchesdurhonensis*’ is a special case. According to the size of the ‘genome’ that represents this species, the dichotomy of both ANI values and the pattern of distinguishing genes and their contamination percentage, the proposed type (and only existing representative) is a mixed culture, presumably of *S. mitis* and *S. pseudopneumoniae,* and thus not a representative of a new species.

The phylogenetic signals reflected in the trees in Figs1^3^ are mutations accumulated in the core genome of the strains. * S. pneumoniae* clones frequently import alleles from *S. mitis* and other commensal streptococci, in some strains up to 8% of the genes in the genome. In contrast, lateral transfer of genes within the *S. mitis* population is limited [19]. The accessory genome of *S. mitis* includes several hundred genes that distinguish strains in full accordance with observations for many other bacterial species [43,50, 51]. Among *S. mitis* strains, the accessory genome includes multiple strain-specific truncated and occasionally intact genes that are remnants of the *S. pneumoniae-*like ancestor and a result of the reductive evolution that shaped the commensal *S. mitis* population [19]. This is especially evident in the genomes of strains in the clusters II, III, IV and *S. thalassemiae* (Table 2). Although the *S. mitis* population is phenotypically homogeneous [negative reaction in traditional laboratory tests for aesculin and arginine hydrolysis, urease, extracellular dextran or levan (not the polysaccharide encoded by the Cps operon), inulin, sorbitol and mannitol fermentation and neuraminidase], several accessory genes are variably present in a pattern that supports some of the phylogenetic clusters (Table 2). Most clearly, clusters Ia-1, Ia-2 and Ib are distinguished from clusters IIa, IIb and IIc by multiple genes. As shown in the table, the gene presence pattern of cluster II strains is more similar to that of *S. pneumoniae* than that of cluster I and III strains. The closer evolutionary relationship of cluster II strains to *S. pneumoniae* is supported by the presence and genomic location of the IgA1 protease gene (*zmpA*). While *S. mitis* strains of cluster II carried the *zmpA* gene in the same genomic context as *S. pneumoniae*, the occasional cluster I strains that possess the *zmpA* gene carry it in the same position as strains of *S. oralis* subsp. *oralis* (Table 2). Based on these differences, we propose to recognize cluster II strains (IIa, IIb and IIc) as a subspecies of *S. mitis* with the name *Streptococcus mitis* subsp. *carlssonii.* The clusters Ia-1, Ia-2 and Ib, which include the type of *S. mitis*, then become *S. mitis* subsp. *mitis.* Table 2 shows several genes that differentiate the two subspecies. However, as some strains of cluster II strains are genetically closer to the designated type of the species (and subsp. *mitis*) than occasional strains of cluster I, and vice versa (Fig. S3 and Table S2), we refrain from proposing them as separate species. At present, the two strains of cluster III remain unclassified strains of *S. mitis*. While cluster III strains are closest to cluster Ia-2 strains according to ANI and dDDH values, their gene pattern (Table 2) is closer to cluster IIc strains. Likewise, the strains of the restricted cluster IV in Fig. 3 (SK1080, Nm_65 and DD26) and strain DD28 (cluster VIII) remain unclassified members of *S. mitis.* Being increasingly close to *S. pseudopneumoniae* and *S. thalassemiae* (Fig. 3), the question of the future will be to define the border between the latter two and *S. mitis* if additional strains turn out to keep falling in the seemingly shrinking inter-species space.

Cluster V consists of three strains from two unpublished studies and appears coherent and well-separated from other strains in the collection. We regard this as a candidate separate species, but as our analyses were based on genomes only, we refrain from formal action. We tentatively use the name ‘*Streptococcus paramitis*’ for cluster V strains. Likewise, the clearly distinct strains S61 (cluster VI), 1001175 (cluster VII) and SK643 belong to separate species if they turn out to represent coherent populations of clones.

Some clinical isolates of *S. pneumoniae*, *S. pseudopneumoniae* and *S. mitis* are difficult to distinguish using classical bacteriological phenotypic tests, analyses based on single gene loci including the 16S rRNA gene or single selected genes, ANI or dDDH values and MALDI-TOF. However, this study confirms previous reports that the phylogenetic analysis based on complete or major parts of genomes eliminates ambiguous identifications [2,14, 15, 50, 52]. An alternative method that appears to be valid for the identification of *S. pneumoniae* is the demonstration of a distinctive nucleotide in the 16S rRNA gene [53]. However, short read-based whole-genome sequencing, which is currently the preferred routine method, often misses the complete 16S rRNA gene. Like *S. pneumoniae*, * S. pseudopneumoniae* constitutes a coherent and distinct cluster by phylogenetic analysis, although the misidentification of * S. pseudopneumoniae* strains (and other species as *S. pseudopneumoniae*) by other methods is a frequent problem that causes confusion [2,14] (see below).

A primary goal of taxonomic designations for bacteria is that they are useful for epidemiological, ecological and clinical purposes and exact communication. In case of a need for epidemiological reference, typing by the recently described MLST system [16] is a useful alternative for *S. mitis*, provided that the database is further developed to be more comprehensive.

### A standalone, light, rapid and portable system for initial phylogeny-based assignment of strains of Mitis group streptococci

Comprehensive whole-genome sequence alignments of large collections of strains require substantial computational power and skills that are rarely associated with clinical microbiology. To enable rapid initial assignment of strains to species, subspecies and clusters as defined in this report, we developed a simple standalone, portable system for anyone to use on any computer large or small running any classical operating system (Windows, MacOS or Unix/Linux). The system uses a Docker container [54] to bundle the Mash, FastANI and QuickTree [55] tools (and the environment they require to run) together with the reference set of 102 genomes that were analysed in detail in this study, supplemented with 15 types of validly published and pending neighbouring species. Detailed but very simple instructions are provided for downloading, installing and running the system (https://hub.docker.com/r/admellodocker/streptoquod). Briefly, all that is needed to instal the tool from Docker is to visit the aforementioned link and follow installation instructions for the user’s operating system. It will prompt the user to create on their Desktop (or anywhere desired) an input folder containing new genomes to classify and an empty output folder and run a Streptoquod command, and the system automatically generates a Mash distance (similar to ANI)-based neighbour-joining phylogenetic tree file that users can open in their favourite tree viewer for inspection of the placement of their new query strains. The system also outputs matrices of Mash and ANI distances between all pairs of reference and query genomes (plain text tab-delimited files that can be opened in Excel, for instance).

For instructional purposes, Fig. S4 shows a tree based on a test analysis of three genome assemblies listed as *S. pseudopneumoniae* in the Genbank database. As demonstrated by the position of the three strains in the tree, two are correctly assigned to *S. pseudopneumoniae*, whereas the third (strain 1172, GCA 001068775) is related to *S. infantis* and, thus, incorrectly identified. The not yet validly published ‘*Streptococcus halitosis*’ is part of the *S. oralis* subsp. *tigurinus* cluster and, thus, a later synonym of that taxon. Finally, according to the genetic distances, ‘*Streptococcus timonensis*’ most likely is part of the species *S. infantis*. A study focused on the *S. infantis* population should be performed to confirm this.

To further illustrate the usefulness of the Streptoquod analysis, we examined all genome sequences assigned to *Streptococcus* sp. and *S. pseudopneumoniae*, respectively, in Genbank. Among 128 genome sequences labelled as *Streptococcus* sp., 2 could be assigned to *S. mitis* subsp. *mitis* cluster 1 a-1 (GCA 030406565.1 and GCA 019414935.1), 1 could be assigned to *S. mitis* subsp. *carlssonii* cluster IIa (GCA 019414905.1), 6 could be assigned to *S. oralis* subsp. *oralis* (GCA 032594295.1, GCA 032500665.1, GCA 030528045.1, GCA 032552255.1, GCA 032574985.1 and GCA 032583915.1), 1 to *S. oralis* subsp. *tigurinus* (GCA 032567935.1) and 1 to *S. oralis* genomosubsp. 1 (GCA 015256435.1). Among 122 genome sequences labelled as * S. pseudopneumoniae*, a total of 25 genomes were incorrectly identified. Two of these could be assigned to *S. mitis* subsp. *mitis* cluster 1 a-1 (GCA 001076775.1 and GCA 001070815); eight could be assigned to *S. mitis* subsp. *mitis* cluster Ia-2 (GCA 001072115.1, GCA 001070805.1, GCA 001072665.1, GCA 001068835.1, GCA 001071735.1, GCA 001070405.1, GCA 001069475.1 and GCA 001068945.1); one could be assigned to *S. mitis* cluster IV (GCA 001070715.1); three could be assigned to *S. mitis* subsp. *carlssonii* cluster IIa (GCA 019414905.1), IIb (GCA 001076615.1) and IIc (GCA 001074825.1), respectively; one could be assigned to the tentative candidate species ‘*S. paramitis*’ (GCA 001069865.1) and two to *S. oralis* subsp*. oralis* (GCA 001068925.1) and *S. oralis* genomosubsp. (GCA 001074565.1), respectively. According to the analysis, the remaining misidentified genome sequences belonged to *S. infantis.*

## Conclusion

A primary goal of taxonomic designations for bacteria is to be useful for epidemiological, ecological and clinical purposes and exact communication. Our study shows that bacteria referred to as *S. mitis* and several proposed new names constitute a continuum of distinct clones. The special evolutionary history combined with the ecological characteristics of the group appears to have shaped this phylogenetic pattern. As virtually every isolate is genetically unique, the likelihood of re-isolating clones is minimal. To recognize most clones as separate species is biologically and practically meaningless. We recommend bending the generally accepted borders to accommodate the biological reality of this group of bacteria. The special population structure of the *S. mitis–S. pneumoniae–S. pseudopneumoniae–S. thalassemiae* complex renders automated classification of isolates based on similarities expressed as ANI or dDDH values to type strains of existing *Streptococcus* species problematic. As an alternative, we present a whole-genome phylogeny-based method that enables phylogenetic comparison of new isolates in the context of a well-characterized reference collection of 102 strains and 15 types of validly published and pending species assigned to the Mitis/Sanguinis group at large.

According to the results of this study, we propose *S. toyakuensis* Wajima *et al*. 2022 [56], *S. chosunensis* corrig. Lim *et al*. 2024 [57], *S. gwangjuensis* corrig. Park *et al*. 2024 [57], *S. humanilactis* Guo *et al.* 2024 [57] and *S. hohhotensis* Li *et al.* 2024 [42] as later heterotypic synonyms of *Streptococcus mitis* Andrewes and Horder 1906 (Approved Lists 1980). The recently reported species *S. parapneumoniae* Katayama *et al.* [58] and *S. nakanonensis* Wajima *et al.* 2025 [58] are proposed as later heterotypic synonyms of *S. thalassemiae* Diouf *et al.* 2023 [59]. The species *S. downii* Martinez-Lamas *et al*. 2020 is proposed as a later heterotypic synonym of *S. oralis* subsp. *dentisani *(Camelo-Castillo *et al*. 2014) Jensen *et al*. 2016. The type strains representing ‘*S. shenyangsis*’, ‘*S. symci*’ and ‘*S. vulneris*’ belong in *S. mitis*. Likewise, the type of ‘*S. halitosis*’ belongs in * S. oralis* subsp. *tigurinus.* Thus, the validation of these species is not supported by our findings. The genome data of the type and only representative of ‘*S. bouchesdurhonensis*’ show that it is a mixed culture.

## Description of two subspecies of *Streptococcus mitis*

*S. mitis* previously included two biovars, 1 and 2 [60]. The subsequently proposed *Streptococcus dentisani* [61] is identical to ‘*S. mitis* biovar 2’. Genetically based phylogenetic studies demonstrated its relationship to *S. oralis,* and the taxon was subsequently reclassified as *S. oralis* subsp*. dentisani* [2]. Consequently, *S. mitis* is currently recognized to be identical to the former ‘*Streptococcus mitis* biovar 1’ [60].

## Emended description of *Streptococcus mitis*

*Streptococcus mitis* (mi’tis. L. masc. adj. *mitis*, mild).

Cells are Gram-positive cocci and grow in short or long chains in serum broth. They are non-spore-forming, aerobic and facultatively anaerobic, non-motile, fermentative and catalase-negative and show *α*-haemolysis on horse blood agar and pronounced greening on chocolate agar due to the production of hydrogen peroxide. The cell wall contains ribitol teichoic acid and lacks significant amounts of rhamnose. The peptidoglycan type is Lys-­direct. Strains are negative in tests for aesculin and arginine hydrolysis and extracellular polysaccharides in the form of dextran or levan. Most strains produce a capsular polysaccharide synthesized by the Wzy/Wzx pathway, in some strains identical to recognized serotypes of *S. pneumoniae* [18]. Strains ferment glucose and sucrose but do not ferment inulin, lactose, sorbitol or mannitol and do not express neuraminidase. The mean genome size is 2 010 000 nt (range 1 800 000–2 215 000).

The species includes two subspecies, *S. mitis* subsp. *mitis* and *S. mitis* subsp. *carlssonii*, in addition to clones (clusters III, IV and VIII) that may represent distinct subpopulations of the species. Genes that distinguish *S. mitis* subsp. *mitis* from *S. mitis* subsp. *carlssonii* are shown in Table 2. Phylogenetic reconstruction distinguishes subclusters Ia-1, Ia-2 and Ib in * S. mitis* subsp. *mitis*. The G+C content of the DNA is ~40%. Found in human mouths and pharynges. The type strain of *S. mitis* and *S. mitis* subsp. *mitis* is NCTC 12261 (ATCC 49456; Carlsson’s strain NS 51; Kilian’s strain SK142; CCUG 31611; CCUG 35790; CIP 103335; DSM 12643; JCM 12971; LMG 14557); 16S rRNA gene sequence accession number AF003929; gap-free genome sequence NZ_CP028414.

## Description of *Streptococcus mitis* subsp*. mitis* subsp. nov.

*Streptococcus mitis* subsp*. mitis* (mi’tis. L. masc. adj. *mitis*, mild).

Cells are Gram-positive cocci and grow in short or long chains in serum broth. They are non-spore-forming, aerobic and facultatively anaerobic, non-motile, fermentative and catalase-negative and show *α*-haemolysis on horse blood agar and pronounced greening on chocolate agar due to the production of hydrogen peroxide. The cell wall contains ribitol teichoic acid and lacks significant amounts of rhamnose. The peptidoglycan type is Lys-­direct. Strains are negative in tests for aesculin and arginine hydrolysis and extracellular polysaccharides. Most strains produce a capsular polysaccharide synthesized by the Wzy/Wzx pathway, in some strains identical to recognized serotypes of *S. pneumoniae* [17]. Strains ferment glucose and sucrose but do not ferment inulin, sorbitol or mannitol and do not express neuraminidase. Some strains ferment lactose (clusters IIb and IIc). Genes that distinguish subsp. *mitis* from subsp. *carlssonii* are shown in Table 2. Phylogenetic reconstruction distinguishes subclusters Ia-1, Ia-2 and Ib. The G+C content of the DNA is ~40%. Found in human mouths and pharynges. The type strain of *S. mitis* subsp. *mitis* is NCTC 12261 (ATCC 49456; Carlsson’s strain NS 51; Kilian’s strain SK142; CCUG 31611; CCUG 35790; CIP 103335; DSM 12643; JCM 12971; LMG 14557); 16S rRNA gene sequence accession number AF003929; gap-free genome sequence NZ_CP028414.

## Description of *Streptococcus mitis* subsp*. carlssonii* subsp. nov.

*Streptococcus mitis* subsp*. carlssonii* (carls.son’i.i. N.L. gen. n. *carlssonii*, of Carlsson, in honour of Swedish oral microbiologist Jan Carlsson, who pioneered ecological and physiological studies of oral streptococci).

Cells are Gram-positive cocci and grow in short or long chains in serum broth. They are non-spore-forming, aerobic and facultatively anaerobic, non-motile, fermentative and catalase-negative and show *α*-haemolysis on horse blood agar and pronounced greening on chocolate agar due to the production of hydrogen peroxide. The cell wall contains ribitol teichoic acid and lacks significant amounts of rhamnose. The peptidoglycan type is Lys­-direct. Strains are negative in tests for aesculin and arginine hydrolysis and extracellular polysaccharides. Most strains produce a capsular polysaccharide synthesized by the Wzy/Wzx pathway, in some strains identical to recognized serotypes of *S. pneumoniae* [17]. Strains ferment glucose and sucrose but do not ferment inulin, sorbitol or mannitol and do not express neuraminidase. Some strains ferment lactose (clusters IIb and IIc). Genes that distinguish subsp. *mitis* from subsp. *carlssonii* are shown in Table 2. Phylogenetic reconstruction distinguishes subclusters IIa, IIb and IIc. The G+C content of the DNA is ~40%. Found in human mouths and pharynges. The type of *S. mitis* subsp. *carlssonii* is strain SK608 (CCUG 55085; LMG 33510); 16S rRNA gene sequence accession number PP270279; genome sequence GenBank JPFZ00000000.1.

## Supplementary material

10.1099/ijsem.0.006704 Uncited Supplementary Material 1.

10.1099/ijsem.0.006704 Uncited Supplementary Material 2.

## Abbreviations

ANI: average nucleotide identity
CDS: coding genes
Cps: capsular polysaccharide synthesis
dDDH: digital DNA–DNA hybridization
MLST: multilocus sequence typing
