# Supplementary material for: Re-evaluation of boundaries of Streptococcus mitis and Streptococcus oralis and demonstration of multiple later synonyms of Streptococcus mitis, Streptococcus oralis and Streptococcus thalassemiae: description of Streptococcus mitis subsp. carlssonii subsp. nov. and emended description of Streptococcus mitis
Source: Int J Syst Evol Microbiol. 2025 Mar 11;75(3):006704. doi: 10.1099/ijsem.0.006704 (PMC12451636; doi:10.1099/ijsem.0.006704)

Re-evaluation of boundaries of *Streptococcus mitis* and *Streptococcus oralis* and demonstration of multiple later synonyms of *Streptococcus mitis*, *Streptococcus oralis*, and *Streptococcus thalassemiae*. Description of *Streptococcus mitis* subsp. *carlssonii* subsp. nov. and emended description of *Streptococcus mitis*.

Mogens Kilian*, Hans-Christian Slotved, Kurt Fuursted, Adonis D’Mello, and Hervé Tettelin

*Corresponding author: Mogens Kilian, kilian@biomed.au.dk

Supplementary figures S1 - S4

Fig. S1. Neighbor-joining tree of 266 genomes constructed in MEGA 11 based on a Mash ANI-like pairwise distance matrix. The tree reveals 14 strains listed as S. mitis in the Genbank database that, according to their position in the tree, belong to other well-established taxa (able S1). In addition, based on database information, 49 genomes represented re-sequencing or re-annotations of the same strain. The type strain of S. mitis NCTC 12261/SK142 alone is represented by six entries in the Genbank database. The analysis further revealed several pairs or groups of up to 23 identical genome sequences. Without exception, these represent isolates from the same sample or groups of 2, 6, or 23 genomes of isolates from genitals of a single couple of sexual partners and represent repeated analyses of the same clone [37]. On the right, the strains in the tree are tentatively assigned to species, subspecies and clusters according to the result of the present study.

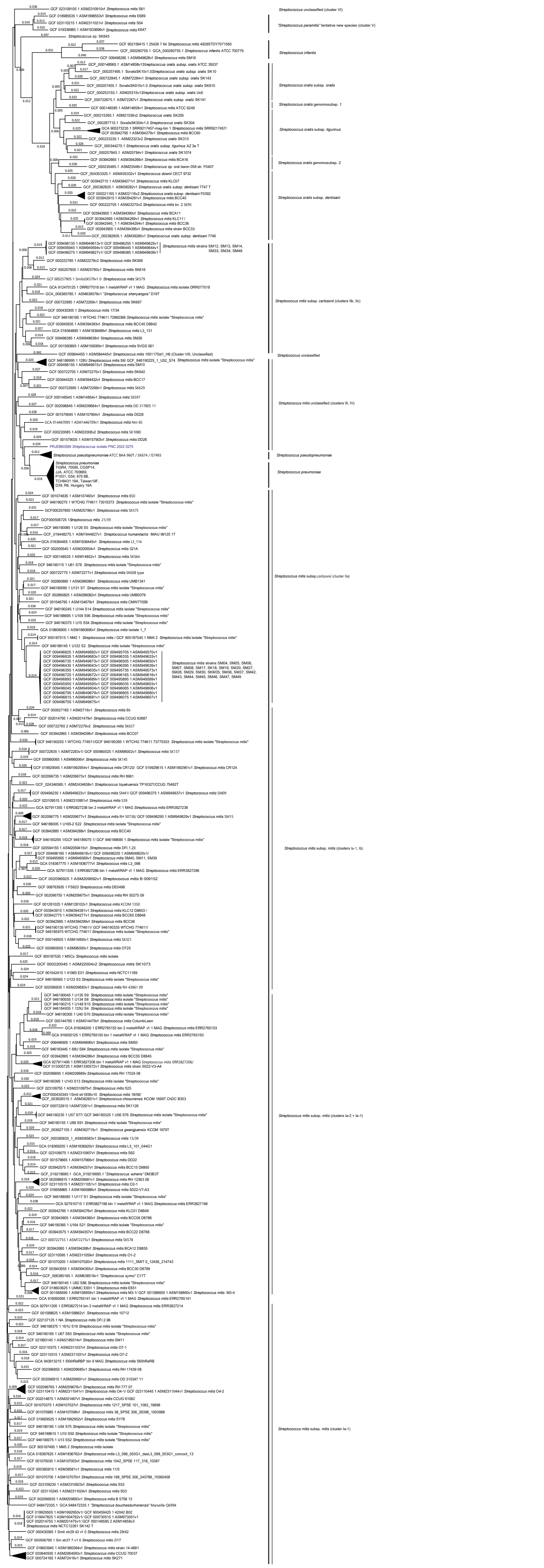

Fig. S2. Ranking of dDDH (d6) values of strains compared to S. mitis NCTC 12261T and the designated type of cluster II (Streptococcus mitis subsp. carlssonii), strain SK608, respectively.

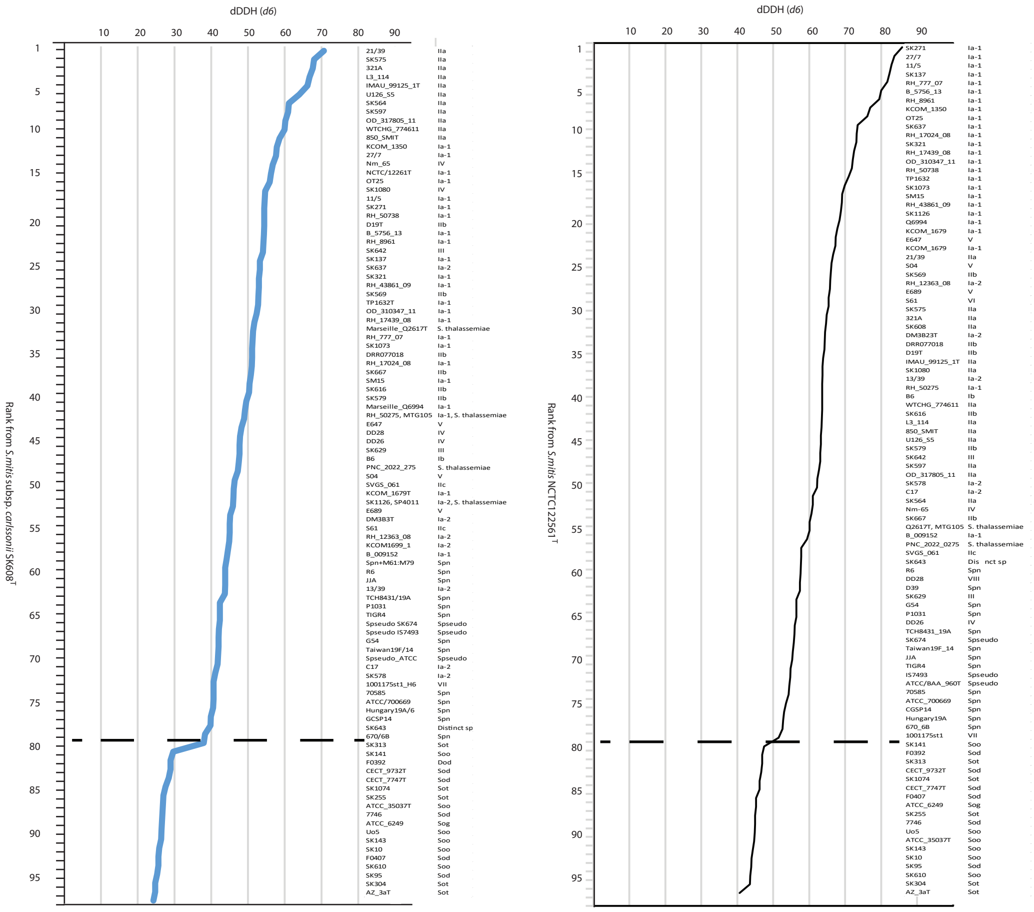

Fig. S3. Spearman correlation plots of all-vs-all pairwise comparisons of genomic similarity estimates (FastANI, Mash distances, and dDDH). P-values are identical as they are extremely low, beyond computational limits. Strong negative correlation with Mash distances is expected as greater genomic similarity implies a small genetic distance and vice versa. A) Correlation of all-vs-all FastANI values versus Mash distances for 100 genomes. B) Correlation of all-vs-all FastANI values versus Mash distance for 270 all-vs-all Mash distances versus dDDH values for 100 genomes.

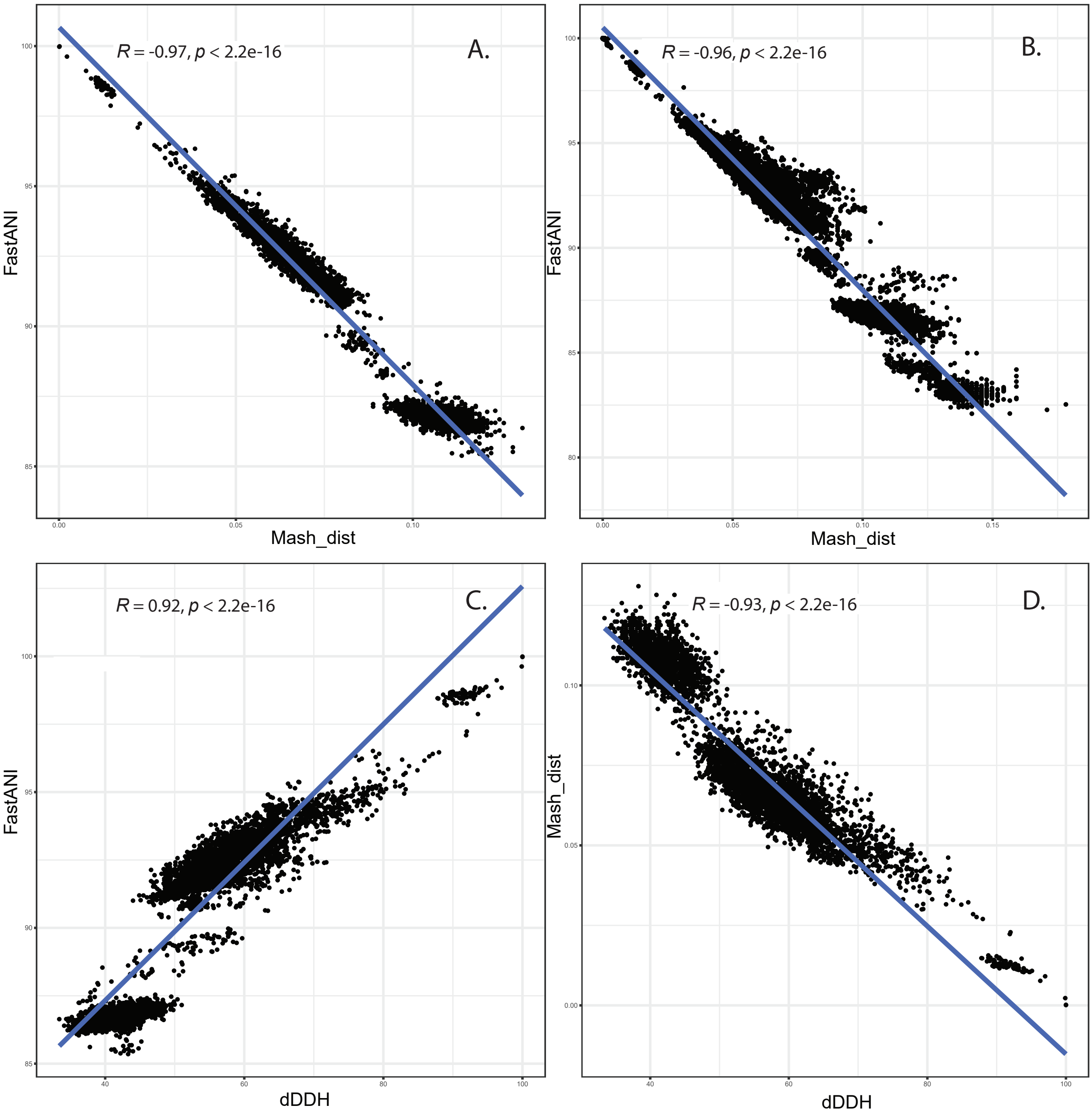

Fig. S4. Phylogenetic tree based on pairwise Mash (ANI-like) distances between each pair of genomes generated by the Streptoquod analysis. The analysis presented in the tree is a test of the Streptoquod 117 default genome sequences compared to three genome assemblies listed as *S. pseudopneumoniae* in the Genbank database. As demonstrated by the position of the three strains in the tree, two (indicated by green dots) are correctly assigned to *S. pseudopneumoniae* whereas the third (strain 1172, GCA 001068775, indicated by red dot) is related to *S. infantis* and, thus, incorrectly identified. The results further demonstrate that the designated type of the proposed species "*Streptococcus halitosis*" is part of the *S. oralis* subsp. *tigurinus* cluster and, thus, a later synonym of that taxon. Likewise, the not validated "*Streptococcus korensis*" appears to be a later synonym of *Streptococcus ilei.* The figures in the tree represent individual branch lengths and the scale at the bottom is the distance scale.

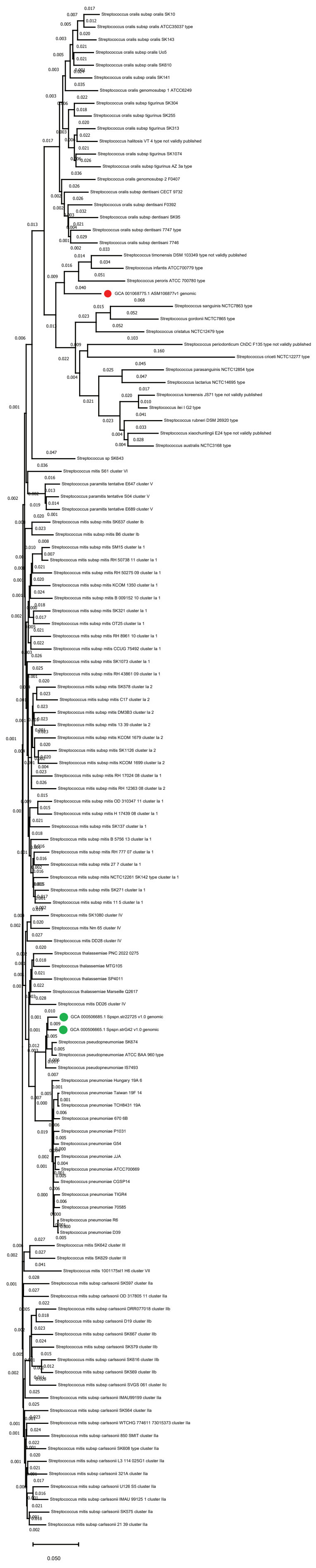

Supplement: Uncited Supplementary Material 1. [file ijsem-75-06704-s001.pdf]
